# Supplementary material for: Drivers and effects of fish-for-sex related single parenthood in a fishing coastal community in Ghana
Source: PLoS One. 2025 Jun 26;20(6):e0325440. doi: 10.1371/journal.pone.0325440 (PMC12200835; doi:10.1371/journal.pone.0325440)
Supplement: S1 Appendix — (DOCX) [file pone.0325440.s001.docx]

## **S1 APPENDIX: QUESTIONNAIRE FOR FISHERS**

Thank you for agreeing to be interviewed today, I appreciate it. My name is *Sylvester Kyei-Gyamfi*. I am a PhD student of the Centre for Migration Studies, University of Ghana, Legon – Accra. I am carrying out this field research work as part of academic work for my Doctorate Degree. The research topic is on “*Mobility and HIV Risk among Fishers in Elmina Fishing Community in the Central Region of Ghana’*. This is will be a question and answer session concerning your movement from your work, movements, settlement conditions, knowledge, attitudes and practices concerning HIV. I will be grateful if you allow me to take a few minutes of your time to engage you in this session. You are assured that any information provided will be treated confidential and used solely for academic purposes only. Please answer all questions openly and honestly. Remember there is right or wrong answer. I am interested in your own personal experiences and opinions. If you want to skip any questions you are free to do so but it is really important that you answer as many as possible. If you do not understand any of the questions just let me know and I will try to explain. Thank you.

**IDENTIFICATION**

| ID1 | Questionnaire ID Number:……………….. | Place of interview…………………………… |  |
| --- | --- | --- | --- |
| ID2 | Telephone number:................................ | Whatsapp number………………………… |  |

**SECTION A: SOCIO-DEMOGRAPHIC BACKGROUND**

To start I would like to ask some questions about yourself:

| DB1 | How old are you today? | 1. <25 2. 25-34 3.45-544.55-645.65-74 6.75+99. Don’t Know |  |
| --- | --- | --- | --- |
| DB2 | What is your sex? | 1. Male 2. Female |  |
| DB3 | What is your highest educational level achieved? | 1.Never been to school 2. Primary3. JSS/JHS  4.Middle School 5.SSS/SHS 5. Vocational/ Technical/ Commercial 6.Post-Middle/Post-Secondary Cert  7. Post-Secondary Diploma  8.Post Graduate 9. Other  |  |
| DB4 | What is your religion? | 1. Islam 2.African Traditionalist3. No religion  4. Christianity 5. Other (Specify)  |  |
| DB5 | Marital status | 1.Single  2.Cohabiting/informal/Consensual  3. Married  4. Divorced  5. Separated 6.Widowed  |  |
| DB6 | Which type of fishing activity do you engagein? | 1. Fish Catching 2. Post-harvesting 3. Maintenance and Repair  4.Porters and Errands  5. Other (Specify)  |  |
| DB7 | How long have you being engaged in this activity in this community? | ………………………………………………. |  |
| DB8 | Nationality | 1.Ghanaian   2. Nigerian   3. Other ECOWAS states (specify) ……………  4.Africa, other than ECOWAS States (specify) ……..  5. European (specify) ………  6.Asian ……………  7. Americas (North, South Caribbean) (specify) ………..  8. Oceania (specify)  …………………………… | **»**→ DB9  **»**→ MS1  **»**→ MS1  **»**→ MS1  **»**→ MS1  **»**→ MS1  **»**→ MS1  **»**→ MS1 |
| DB9 | Which ethnic group do you belong to? | 1.Fante 2.Other Akan  3.Ga Adangbe  4.Mole Dagbani 5.Ewe  6.Guan  7.Other (specify) ……  99. Don’t know  |  |

**SECTION 2: MOBILITY AND SETTLEMENT PATTERNS AND HIV RISKS**

Now I would like to ask you some questions about mobility and settlement patterns. This will involve questions on the reasons that account for your mobility to other fishing community, and the conditions your settlement in the fishing destination points you visited in the last 12 months

| MS1 | Where is your current place of residence? | ……………………………………… |  |
| --- | --- | --- | --- |
| MS2 | How long have you lived/been in this fishing community? | ……………………………………… |  |
| MS3 | Do you travel to other fishing destinations to engage in fishing related activities | 1.Yes  2. No  | IF ‘NO’ **»**→ AK1 |
| MS4 | In the last 12 months have travelled to other fishing destination to engage in any fishing related activity? | 1.Yes  2. No  | IF ‘NO’ **»**→ MS7 |
| MS5 | If yes, what was the main reason for travelling to other fishing destinations | 1.To buy or sell fish 2.To serve as fish potter 3. To assist Family member’s fishing activity 4. To track fish in other fishing communities  5. To do repair or maintenance work on boats 6. Other {Specify}.. |  |
| MS6 | How many times have you travelled away from this village in the last 12 months? | 1.Once  2.Twice  3.Three times  4. Four times  5. Five times  6. More than five times |  |
| MS7 | How long do you usually stay when you are in other fishing communities? | 1. Day 2. Week 3. Month 4. More than a month 5. Other {Specify}…  |  |
| MS8 | What are the reasons for staying (keeping you) in this/these communities? | ……………………………………… |  |
| MS9 | Does staying away from home affect you in any way? | 1.Yes  2. No  | IF ‘NO’ **»**→ MS11 |
| MS10 | How does staying away from home affect you? | 1. Influences non-regular sexual behaviours  2. Leads to loneliness and isolation  3. Leads to the indulgence in alcohol abuse  4. Other {Specify}… |  |
| MS11 | Where do you normally reside in the other destinations they travel to? | 1. Makeshift wooden structures 2.In friends/relatives home 3. Rented rooms around the fish village 4.Guest house 5.On the boats/canoe |  |
| MS12 | Do you face any other challenges settling in these other fishing communities? | 1.Yes  2. No  | IF ‘NO’ **»**→ AK1 |
| MS13 | If yes, what are these challenges? | ……………………………………….. |  |

**SECTION 4: HIV AND AIDS RELATED KNOWLEDGE AND ATTITUDES OF FISHERS**

I would like to you some few questions about your awareness and knowledge on HIV/STI, and remember there is no right or wrong answer.

| AK1 | Are you aware of Sexually Transmitted Infections (STIs)? | 1.Yes  2. No  |  |
| --- | --- | --- | --- |
| AK2 | Have you ever heard of HIV and/or AIDS? | 1.Yes  2. No  | IF ‘NO’ **»**→ RF1 |
| **Knowledge of HIV Prevention Methods** | | | |
| AK3 | Is there a particular way of protecting oneself from HIV? | 1.Yes  2. No  | IF ‘NO’ **»**→ RF1 |
| AK4 | Indicate the protective method of HIV | A. Using condoms 1.Yes  2. No   B. Abstinence/ Being faithful 1.Yes  2. No   C. Limiting sex to one uninfected partner1.Yes  2. No   D. Avoiding sharing of sharp/piercing objects1.Yes  2. No  | Multiple Response |
| **Sources of Knowledge on HIV and AIDS** | | |  |
| AK5 | What is your source of information on HIV and/or AIDS? | A.Television1.Yes  2. No   B. Radio1.Yes  2. No   C. Newspaper1.Yes  2. No   D. Pamphlet/posters1.Yes  2. No   E. Health Care workers1.Yes  2. No   F. Religious leader1.Yes  2. No   G. Traditional leader1.Yes  2. No   H. Campaign1.Yes  2. No   I. Family member1.Yes  2. No   J. Friend1.Yes  2. No   K. Workplace 1.Yes  2. No   L. Sexual partner 1.Yes  2. No  | Multiple Response |
| AK6 | What is your main source of information on HIV and AIDS? | 1.Television 2.Radio 3. Newspaper 4. Pamphlet/ posters 5. Health Care workers  6. Religious/Traditional leader  7. Community Durbar/Townhall gathering   8. Family member  9. Friend10. Workplace . |  |
| **Comprehensive Knowledge of HIV and AIDS** | | | |
| AK7 | Can a healthy looking person have HIV? | 1.Yes  2. No  |  |
| AK8 | Can you become infected with HIV and AIDS having sexual intercourse without a condom? | 1.Yes  2. No  |  |
| AK9 | Can you become infected with HIV and AIDS from mosquito bites? | 1.Yes  2. No  |  |
| AK10 | Can you become infected with HIV and AIDS through witchcraft? | 1.Yes  2. No  |  |
| AK11 | Can the risk of HIV infection be reduced by having sexual intercourse with only one faithful uninfected partner? | 1.Yes  2. No  |  |
| AK12 | Is AIDS a dangerous disease? | 1.Yes  2. No  |  |
| AK13 | Explain your answer in ??? | ……………………………………… |  |
| AK14 | Are you afraid of AIDS? | 1.Yes  2. No  |  |
| AK15 | Explain your answer | …………………………. |  |
| AK16 | Have you ever had an HIV test? | 1.Yes  2. No  |  |
| AK17 | Would you like to have an HIV test? | 1.Yes  2. No  | IF ‘YES’ **»**→ AK19 |
| AK18 | If no, why would you not want to be tested? | ……………………………………… |  |
| AK19 | If yes, do you know where to have an HIV test? | 1.Yes  2. No  |  |

**SECTION 5: RISKY SEXUAL BEHAVIOURS THAT EXPOSE FISHERS TO HIV**

Now I want us to talk about your sexual activity in the last 12 months; whether you have had sex, whether you have had any sexual encounter (s) with any non-regular partner and the number of such partners. I would also ask you questions about condoms use in your sexual encounters within the period.

| RF1 | Have you had sex in the last 12 months? | 1.Yes  2. No |  |
| --- | --- | --- | --- |
| RF2 | Have you had sexual intercourse with any non-regular partner in the last 12 months? | 1.Yes  2. No  | IF ‘NO’ **»**→ RF4 |
| RF3 | If yes, about how many non-regular partners have you had intercourse with in the last 12 months | 1. One partner 2. Two partners 3. Three partners or more |  |
| RF4 | Have you been using condoms in the last 12 months during sexual intercourse? | 1.Yes  2. No  | IF ‘NO’ **»**→ RF7 |
| RF5 | Was condom used the last time you had sex with a non-regular partner? | 1.Yes  2. No  |  |
| RF6 | If yes, how often? | 1. Always 2. Not always 3. Occasionally 4. Never |  |
| RF7 | IF no what was the main reason for not using a condom the last time you had sex with a non-regular partner? | 1. Partner objected to the use of condoms 2. Condom was not available 3. Trust partner/Didn’t think it was necessary 4. Attractiveness of partner |  |
| RF8 | Have you heard of Fish-for-sex (FFS) transactional relationship? | 1.Yes  2. No  | IF ‘NO’ **»**→ IP1 |
| RF9 | Have you ever engaged in Fish for sex? | 1.Yes  2. No  |  |
| RF10 | In the last 12 months have you engaged in FFS? | 1.Yes  2. No  |  |
| RF11 | Do you use a condom when you have FFS sex? | 1.Yes  2. No  |  |
| RF12 | If yes, what is the main reason for using a condom in FFS sex? | 1.Condom reduces pleasure of sex 2. My partner does not like to use condoms 3. Trust for partner/Didn’t think it was necessary 4. Condom not available |  |
| RF13 | Did you use a condom the last time you had a FFS sex? | 1.Yes  2. No  |  |
| RF14 | If yes what was the main reason for not using a condom the last time you had FFS sex? | ………………………………………………………………. |  |
| RF15 | Why do you engage in FFS transactional relationship? | ………………………………………………………………. |  |
| RF16 | Have you ever encountered stories or reports about single parenthood resulting from FFS relationships in Elmina? | 1.Yes  2. No  |  |
| RF 17 | In your opinion how will you perceive the frequency of occurrence of single FFS parenthood in Elmina? | 1. Never 2. Rarely 3. Sometimes 4. Often 5. Always |  |
| RF 18 | What factors do you believe contribute to the prevalence of FFS relationships in Elmina? | ……………………………………………………………………………… |  |
| RF 19 | What are the perceived effects of single-female parenting? | ……………………………………………………………………………… |  |

**SECTION 6: STRATEGIES FOR CARRYING OUT HIV EDUCATION IN STUDY AREA**

Here I will ask you questions regarding your participation in HIV and AIDS education programmes, reasons for non-participation, the kinds of HIV and AIDS educational interventions currently on-going in the Elmina fishing community and other fishing communities you visited in the last 12 months. This will be very short.

| IP1 | Are you aware of any HIV education in this community? | 1.Yes  2. No  | IF ‘NO’ **»**→ IP3 |
| --- | --- | --- | --- |
| IP2 | If yes, which agency/organisation is implementing this HIV education programme? | …………….. |  |
| IP3 | Have you participated in any HIV programmes in the last 12 months**?** | 1.Yes  2. No  |  |
| IP4 | If you no, why not |  |  |
| IP5 | Have there been any changes in your sexual behaviour due to your participation in any HIV programmes? | 1.Yes  2. No  99. Don’t Know | IF ‘NO’ **END** |
| IP6 | If yes, what changes have occurred in your sexual behaviour due to being part of the HIV program? |  |  |

**Thank you!**
