# Supplementary material for: Drivers and effects of fish-for-sex related single parenthood in a fishing coastal community in Ghana
Source: PLoS One. 2025 Jun 26;20(6):e0325440. doi: 10.1371/journal.pone.0325440 (PMC12200835; doi:10.1371/journal.pone.0325440)
Supplement: S2 Appendix — (DOCX) [file pone.0325440.s002.docx]

## **S2 APPENDIX: INTERVIEW GUIDES FOR PARTICIPANTS**

## **MALE & FEMALE FOCUS GROUP DISCUSSION (FGD) TOOL**

1. **BACKGROUND INFORMATION**
2. Physical aspects
   1. How are the areas in the neighborhood different from others?
   2. What are the patterns of settlement?
   3. Do both fisher migrants and non-migrants, male and female fishers live in same settlement?
   4. How do you access water in Elmina and other fishing communities you travel to?
   5. What kind of toilet facilities do you use in Elmina and in the other places you travel to?
   6. What are your challenges with water, toilet and other sanitation facilities in the fishing communities you know of?
   7. Generally what are the states of infrastructure and amenities in the fishing communities that you have visited? What are the key challenges that fishers face in fishing communities?
3. Demographic profile of the population
   1. Who makes up the community?  Age, gender, ethnicity, marital status etc.
4. Social structure
   1. Community leaders - formal and informal, Community culture, formal and informal, existing groups, existing institutions. Attitudes and values.
5. Economics (*probe to find out ways in which people earn their living in this community*)
   1. What is the main economic activity? What alternative economic activities exist? What do men and women do for a living? What are the gender roles assigned to both male and female in the fishing activity? Are there inequalities? Please explain
   2. What is the main economic activity? What alternative economic activities exist? What do men and women do for a living? What are the gender roles assigned to both male and female in the fishing activity? Are there inequalities? Please explain
6. **MOBILITY**
7. How do female/male fishers organize their mobility to the fishing destinations?
8. Do they travel alone or with other members of their families or close relatives?
9. What are the situations that keep female/male fishers in other fishing destinations?
10. What are the settlement patterns (meals, sleeping arrangements and sanitation issues etc.)
11. What risky lifestyles do female/male fishers engage in the fishing community?
12. Have you heard of fish-for-sex sexual exchanges?
13. Do fishers in Elmina engage in FFS transactions? If yes, what are men/women’s subjective position regarding ‘fish for sex’ exchange?
14. How do members of the community see women and men engaged in FFS?
15. Why do male/female fishers engage in FFS?
16. Do you think FFS can cause HIV among fishers? If yes, why do you say so?
17. Have you ever encountered stories or reports about single parenthood resulting from FFS relationships in Elmina?
18. In your opinion how will you perceive the frequency of occurrence of single FFS parenthood in Elmina?
19. What factors do you believe contribute to the prevalence of FFS relationships in Elmina?
20. What are the perceived effects of single-female parenting?
21. Generally, what are the main challenges of male/female fishers in this community?
22. **Knowledge, Attitudes and Practices Regarding HIV**
23. Is there anyone here who has not heard of HIV?
24. What do you know about HIV and AIDS?
25. What do you think causes AIDS?
26. How do you know if someone has the virus that causes AIDS?
27. How do people get infected?
28. Who is at risk of getting infected with HIV, the virus that causes AIDS? Who is not at risk?
29. In your opinion, what are some of the risky lifestyles in Elmina and the other fishing communities you have visited that might expose fishers to HIV?
30. How important do you think HIV is compared to other diseases people have?
31. What can the people in this community do to keep from getting infected?
32. How easy or difficult is it to do these things?
33. How would people in this area feel if someone in their family or community were infected with HIV?
34. What or who do you think could be good sources of information about AIDS that people would really listen to? Why?
35. What can fisherfolks do to help prevent HIV and AIDS in this community?
36. What can fisherfolks do to help people and the families of people who are already HIV-infected or have AIDS?
37. What sorts of information/training/materials/other support do you need to prevent people from getting infected with HIV and to support people with AIDS and their families?

Are there any other areas you would like to comment on that we haven’t covered today that you think should be included? If not, then I would say thank you for taking part in the focus group discussion.

## **INTERVIEW GUIDE FOR GOVERNMENT INSTITUTIONS (GAC, MHA, FD, DEVELOPMENT AND THE MUNICIPAL ASSEMBLY, DEPARTMENT OF GENDER, AND DEPARTMENT OF COMMUNITY DEVELOPMENT**

**GHANA AIDS COMMISSION (GAC)**

- 1. **Introductory Questions**

I would like to start off with some general questions about your involvement with issues on fishers.

1. Is the Ghana Decentralized? Do you have offices in the Districts?
2. To what extent is GAC involved in community outreach and public education?
3. What are your methods for engaging with the public on HIV and other STIs?
4. How effective are your methods and strategies for carrying out HIV education?
5. What are the major steps taken by the Ghana AIDS Commission to improve HIV prevention in the fishing sector?
6. Does the GAC have any programme that seeks to enhance HIV prevention among fishers?
7. Does the GAC have any programme that seeks to enhance HIV prevention among fishers the community? Can you provide details on this project or programme?
8. When did you first start with this project? What is the duration of the project?
9. How is it related to fisher mobility and HIV exposure?
10. Are you working in only Elmina fishing community or other communities?
11. If you are working in other communities, please briefly share some of your experiences in respect to knowledge, attitudes and practices of fishers regarding HIV.
12. What are your methods for carrying our HIV information to fishers and the fisher community, if any?
13. What are the challenges you face regarding HIV education in the community?
14. How is the project financed
    1. **Achievement and challenges**
15. What has been the biggest achievement of your engagement with fishers concerning HIV education to date? Why did you select that achievement?
16. What has been the biggest challenge your engagement with fishers concerning HIV education to date?
    1. Why did you select that challenge?
    2. How have you coped with the challenges?
17. What do you propose to be done to improve knowledge, attitudes and practices of fishers regarding HIV and other risky behaviours that increase fishers’ exposure to HIV?

Now I would like to talk about human rights, legislations and regulations on HIV.

1. Does the country have laws and regulations that protect people living with HIV against discrimination? (*Such as general non-discrimination provisions or provisions that specifically mention HIV, focus on schooling, housing, employment, health care etc*.)
2. Does the country have non-discrimination laws or regulations which specify protections for vulnerable sub-populations such as fishers?
3. Has the Government, through political and financial support, involved most at-risk populations in governmental HIV-policy design and programme implementation?
4. At present the HIV prevalence rates in fishing communities in Ghana is known. Does the GAC or government have any future plans to improve data on HIV among high prevalent HIV sub-populations such as fishers? Briefly give an account of any such intentions
5. Are there programmes to change societal attitudes of stigmatization associated with HIV and AIDS to understanding and acceptance? If Yes, what types of programmes.

**FISHERIES DEPARTMENT (FD)**

- 1. **Introductory Questions**

I would like to start off with some general questions about your involvement with issues on HIV among fishers.

1. Is HIV having any effects on the fisheries sector? What are the effects and how has it impacted on fisheries management and development?
2. What are the major steps taken by the Fisheries Department to improve Fisheries management and development?
3. Does the Department have any programme that seeks to enhance HIV prevention among fishers? Can you provide details on this project or programme?
   1. When did you first start with this project? What is the duration of the project?
   2. How is it related to fisher mobility and HIV exposure?
   3. Are you working in only Elmina fishing community or other communities?
   4. If you are working in other communities, please briefly share some of your experiences in respect to knowledge, attitudes and practices of fishers regarding HIV.
   5. What are your methods for carrying our HIV information to fishers and the fisher community, if any?
   6. What are the challenges you face regarding HIV education in the community?
   7. How is the project financed
   8. **Achievement and challenges**
4. What has been the biggest achievement of your engagement with fishers concerning HIV education to date? Why did you select that achievement?
5. What has been the biggest challenge your engagement with fishers concerning HIV education to date?
   1. Why did you select that challenge?
   2. How have you coped with the challenges?
6. What do you propose to be done to improve knowledge, attitudes and practices of fishers regarding HIV and other risky behaviours that increase fishers’ exposure to HIV?

**MUNICIPAL HEALTH ADMINISTRATION (MHA)**

- 1. **Introductory Questions**

I would like to start off with some general questions about your involvement with issues on fishers.

- 1. **Awareness, knowledge, attitudes and practices of fishers regarding HIV**

1. Is HIV risk a major concern among fishers in Elmina?
   1. Do the fisherfolks in Elmina consider HIV as a major risk and threat to their development? Please share your thoughts on this.
   2. How would you describe the level of awareness and knowledge of fishers regarding HIV?
   3. What are your observations on the attitudes and practices of fishers regarding HIV in this community and the fishing communities you operate in?
2. What are the major concerns regarding HIV risk behaviours?
   1. Is HIV risk denial an issue here?
   2. Are fishers engaged in excessive alcohol drinking?
   3. Do fishers engage in drugs?
   4. What are the issues concerning fishers and sex networking (casual sex, multiple sexual relationships, Sex with sex workers and fish-for-sex transactions) – (*Take the issues one after the other and probe for details*).
3. Is the Health Directorate currently working on any project concerning the protection of fishers against HIV and other STIs? Is the project offered in this community/and or other communities? Can you provide details on this project or programme?
   1. When did you first start with this project? What is the duration of the project?
   2. How is it related to fisher mobility and HIV exposure?
   3. Are you working in only Elmina fishing community or in other fishing communities?
   4. If you are working in other communities, please briefly share some of your experiences in respect to knowledge, attitudes and practices of fishers regarding HIV.
   5. What are your methods for carrying our HIV information to fishers and the fisher community, if any?
   6. Do fishers from the community visit your service? How accessible are your services (very easy, easy, very difficult, difficult, impossible). What do fishers use your facility for?
   7. What are the challenges you face regarding HIV education in the community?
   8. How is the project financed
4. Do you have incentives in place to ensure safe sex among fishers?
   1. Do you distribute free condoms, or provide free counselling on HIV and STIs.
   2. Do you, or any other local service, provide voluntary counselling and testing services (VCT) in the community? If yes what is the name of the organisation?
   3. **Achievement and challenges**
5. What are the key strengths of your organisation and the service it provides?
6. What difficulties does the institution experience in delivering services or what stops it from meeting the needs of the community? (*Probe: lack of resources, inaccessibility, loss of staff, poverty etc*.)
7. What has been the biggest achievement of your engagement with fishers concerning HIV education to date? Why did you select that achievement?
8. What has been the biggest challenge your engagement with fishers concerning HIV education to date?
   1. Why did you select that challenge?
   2. How have you coped with the challenges?
   3. Is there anything else you would like to tell me about the HIV and AIDS related services you provide in relation to fishing communities, or about the groups of people from fishing communities who use your service and how they are being affected by HIV/AIDS?
9. What do you propose to be done to improve knowledge, attitudes and practices of fishers regarding HIV and other risky behaviours that increase fishers’ exposure to HIV?

**KOMENDA EDINA EGUAFO ABIREM (KEEA) MUNICIPAL ASSEMBLY**

- 1. **Introductory Questions**

I would like to start off with some general questions about your involvement with issues on HIV among fishers.

1. Is HIV a major concern in the Elmina fishing community? Please share your thoughts on it.
2. Do you feel HIV is having any effects on the fisheries sector? What are the effects and how has it impacted on fisheries management and development?
3. Does the Assembly have any programme that seeks to enhance HIV prevention among fishers? Can you provide details on this project or programme?
4. When did you first start with this project? What is the duration of the project?
5. How is it related to fisher mobility and HIV exposure?
6. What are your methods and strategies used by the Assembly for carrying our HIV information to fishers and the fisher community, if any?
7. What are the challenges you face regarding HIV education in the community?
8. Does the Assembly make budgetary provision for HIV programmes in the Assemblies budget? What are your sources of funding for HIV interventions? What percentage of the budget is allocated for HIV programmes? What are the specific activities captured in the budget of the Assembly?
   1. **Achievement and challenges**
9. What has been the biggest achievement of the Assembly’s engagement with fishers concerning HIV education to date? Why did you select that achievement?
10. What has been the biggest challenge your engagement with fishers concerning HIV education to date?
    1. Why did you select that challenge?
    2. How have you coped with the challenges?
11. What do you propose to be done to improve knowledge, attitudes and practices of fishers regarding HIV and other risky behaviours that increase fishers’ exposure to HIV in the KEEA Municipality?

**DEPARTMENT OF COMMUNITY DEVELOPMENT AND DEPARTMENT OF GENDER**

- 1. **Introductory Questions**

I would like to start off with some general questions about your involvement with issues on fishers.

- 1. **Awareness, knowledge, attitudes and practices of fishers regarding HIV**

1. Is HIV risk a major concern among fishers in Elmina?
   1. Do the fisherfolks in Elmina consider HIV as a major risk and threat to their development? Please share your thoughts on this.
   2. How would you describe the level of awareness and knowledge of fishers regarding HIV?
   3. What are your observations on the attitudes and practices of fishers regarding HIV in this community and the fishing communities you operate in?
2. Is the Department of Community Development/Department of Gender currently working on any project concerning the protection of fishers against HIV and other STIs? Is the project offered in this community/and or other communities? Can you provide details on this project or programme?
   1. When did you first start with this project? What is the duration of the project?
   2. How is it related to fisher mobility and HIV exposure?
   3. Are you working in only Elmina fishing community or in other fishing communities?
   4. If you are working in other communities, please briefly share some of your experiences in respect to knowledge, attitudes and practices of fishers regarding HIV.
   5. What are your methods for carrying our HIV information to fishers and the fisher communities, if any?
   6. What are the challenges you face regarding HIV education in the community?
   7. How is the project financed

iii. What is the general attitude toward parenting in Elmina, particularly regarding single parenthood and FFS relationships?

- 1. Have you ever encountered stories or reports about single parenthood resulting from FFS relationships in Elmina?
  2. In your opinion how will you perceive the frequency of occurrence of single FFS parenthood in Elmina?
  3. What factors do you believe contribute to the prevalence of FFS relationships in Elmina?
  4. What are the perceived effects of single-female parenting?
  5. **Achievement and challenges**

1. What are the key strengths of your organisation and the service it provides?
2. What difficulties does the institution experience in delivering services or what stops it from meeting the needs of the community? (*Probe: lack of resources, inaccessibility, loss of staff, poverty etc*.)
3. What has been the biggest achievement of your engagement with fishers concerning HIV education to date? Why did you select that achievement?
4. What has been the biggest challenge your engagement with fishers concerning HIV education to date?
5. What do you propose to be done to improve knowledge, attitudes and practices of fishers regarding HIV and other risky behaviours that increase fishers’ exposure to HIV?

## **INTERVIEW GUIDE FOR NON-GOVERNMENTAL ORGANISATIONS (FOUNDATION FOR BUILDERS AND KIDS CLUBS & HUMAN SERVICE TRUST FOUNDATION)**

- 1. **Introductory Questions**

1. Are you working on any project concerning movements of fishers and their protection against HIV and other STIs? Can you provide details on this project or programme?
   1. When did you first start with this project?
   2. How is it related to fisher mobility and HIV exposure?
   3. Are you operating in only Elmina fishing community or in other fishing communities?
   4. If you are working in other fishing communities, please briefly share some of your experiences in respect to knowledge, attitudes and practices of fishers regarding HIV.

**b. Awareness, knowledge, attitudes and practices of fishers regarding HIV**

1. Is HIV risk a major concern among fishers in Elmina?
   1. Do the fisherfolks in Elmina consider HIV as a major risk and threat to their development? Please share your thoughts on this.
   2. How would you describe the level of awareness and knowledge of fishers regarding HIV?
   3. What are your observations on the attitudes and practices of fishers regarding HIV in this community?
2. What are the major concerns regarding HIV risk behaviours?
   1. Is HIV risk denial an issue here?
   2. Are fishers engaged in excessive alcohol drinking?
   3. Do fishers engage in drugs?
   4. What are the issues concerning fishers and sex networking (casual sex, multiple sexual relationships, Sex with sex workers and fish-for-sex transactions) – (*Take the issues one after the other and probe for details*).

iv. What is the general attitude toward parenting in Elmina, particularly regarding single parenthood and FFS relationships?

a. Have you ever encountered stories or reports about single parenthood resulting from FFS relationships in Elmina?

b. In your opinion how will you perceive the frequency of occurrence of single FFS parenthood in Elmina?

c. What factors do you believe contribute to the prevalence of FFS relationships in Elmina?

d. What are the perceived effects of single-female parenting?

v. What are your methods for carrying our HIV information to fishers and the fisher community, if any?

- 1. What are the challenges you face regarding HIV education in the community?
  2. How have you coped with the challenges?
  3. What have been your success stories in your work with the fishers?
  4. What will you recommend to enhance your work?

1. Do you have incentives in place to ensure safe sex among fishers?
   1. Do you distribute free condoms, or provide free counselling on HIV and STIs.
   2. What other incentives do you have?
   3. **Achievement and challenges**
2. What has been the biggest achievement of your engagement with fishers concerning HIV education to date? Why did you select that achievement?
3. What has been the biggest challenge your engagement with fishers concerning HIV education to date? Why did you select that challenge?
4. What do you propose to be done to improve knowledge, attitudes and practices of fishers regarding HIV and other risky behaviours that increase fishers’ exposure to HIV?

## **INTERVIEW GUIDE FOR KEY INFORMANT INTERVIEWS COMMUNITY LEADERS (OPINION LEADERS)**

- 1. **Fisher mobility and settlement**

1. How long have you lived in this community? Please kindly tell me about the fishing activity in this area?
2. Why do fishers move to fish in this area, and where from?
   1. Why do they leave and where do they go to?
   2. Since when have they been migrating to this community?
   3. How is their migration organised? Do they migrate alone, with families or in groups?
   4. What position do they have in the receiving settlement?
   5. How do they get permission to stay and access to the fishing grounds?

Now I will like us to talk about fishers in this community and their knowledge, attitudes and practices regarding HIV.

- 1. **Awareness, knowledge, attitudes and practices of fishers regarding HIV**

1. Is HIV risk a major concern among fishers in Elmina?
   1. Do the fisherfolks in Elmina consider HIV as a major risk and threat to their development? Please share your thoughts on this.
   2. How would you describe the level of awareness and knowledge of fishers regarding HIV?
   3. What are your observations on the attitudes and practices of fishers regarding HIV in this community?
   4. How do fisherfolks and members of this community get their information on HIV? Are these sources effective in providing adequate information on the disease?
   5. What have been your overall impressions of HIV education in the community?
   6. Has your own knowledge, attitudes or practices on HIV changed since you heard of HIV? If yes:
      1. What has changed in terms of your knowledge, attitudes or practices?
      2. What do you think caused these changes to happen?
      3. Can you say same of other members of the community?
2. What are the major concerns regarding HIV risk behaviours?
   1. Is HIV risk denial an issue here? Do you think fishers ignore the danger posed by HIV due to the daily dangers they face at sea?
   2. Are fishers engaged in excessive alcohol drinking? Do fishers engage in drugs?
   3. What are the issues concerning fishers and sex networking (casual sex, multiple sexual relationships, Sex with sex workers and fish-for-sex transactions) – (*Take the issues one after the other and probe for details*)
   4. What are people’s subjective position regarding ‘fish for sex’ exchange? What do community members feel about FFS practice? How do members of the community see women and men engaged in FFS? Is it likely women who engage in FFS could face social exclusion or stigmatization?
   5. Have you observed any changes in sexual behaviours since the outbreak of HIV?
   6. Is HIV one of the factors that have contributed to the changes?
   7. What changes in sexual behaviours have you observed in the community since the outbreak of HIV? What actions caused these changes?

v. What is the general attitude toward parenting in Elmina, particularly regarding single parenthood and FFS relationships?

a. Have you ever encountered stories or reports about single parenthood resulting from FFS relationships in Elmina?

b. In your opinion how will you perceive the frequency of occurrence of single FFS parenthood in Elmina?

c. What factors do you believe contribute to the prevalence of FFS relationships in Elmina?

d. What are the perceived effects of single-female parenting?
