# Supplementary material for: Drivers and effects of fish-for-sex related single parenthood in a fishing coastal community in Ghana
Source: PLoS One. 2025 Jun 26;20(6):e0325440. doi: 10.1371/journal.pone.0325440 (PMC12200835; doi:10.1371/journal.pone.0325440)
Supplement: S3 Appendix — (PDF) [file pone.0325440.s003.pdf]

S3 APPENDIX: PROTOCOL CONSENT FORM

UNIVERSITY OF GHANA

Official Use only
Protocol number

OFFICE OF RESEARCH, INNOVATION AND DEVELOPMENT

Ethics Committee for Humanities (ECH)

PROTOCOL CONSENT FORM

Title of Study	Mobility and HIV Risk among Fishers in Elmina Fishing Community in the Central Region of Ghana
Principal Investigator	Sylvester Kyei-Gyamfi
Certified Protocol Number	ECH 118/16-17

General Information about Research

The study titled 'Mobility and HIV Risk among Fishers in Elmina Fishing Community in the Central Region of Ghana' is being undertaken in partial fulfilment of the requirement for the award of PhD in Migration Studies at the Centre for Migration Studies of University of Ghana, Legon-Accra. The aim of this study is to establish the relationship between mobility of fishers and the risk of HIV infection. The specific objectives are to describe mobility and settlement patterns of fishers, assess their knowledge, attitudes and practices regarding HIV, explore HIV risk factors associated with mobility, examine the gender differences in HIV risk exposure among fishers, and investigate the impacts of the approaches used in carrying out HIV information in the study area. Elmina was chosen as the study area using convenience sampling method taking into account the under mentioned reasons as basis:

i. Elmina being the fishing community with the largest number of fisher populations in the Central Region (Aheto et al., 2012; Korankye, 2008; Korankye and Dwomoh, 2012) and as a result will be appropriate for easy access to fisher populations.

ii. Elmina having access to a berthing and landing facilities for both inshore vessels and canoes, which serve as a hub of fishers from across the country and the other countries within the West African sub-region to engage in fishing and other related activities (KEEA, 2014), making it suitable for on-site and direct physical observation of settlement patterns, states of social infrastructure and services (schools, hospitals etc.), sleeping and living arrangements, the repair, moulding and making of fishing gears, and the general experience of fishers.

iii. Elmina having a number of Fisher Associations and Fish Monger Associations that will be very useful in the sampling design.

The study will utilize a structured questionnaire for the quantitative aspects (survey), an interview guide for the key informant interviews and focus group discussions for the qualitative aspects. The interviews for the survey will take between 45 minutes and 1 hour to complete, the Key Informant Interviews and focus group discussions will also take between 1 hour and 2 hours per session. Owing to the lengthy nature of the instruments, the researcher will allow participants to have short interval breaks (15-20 minutes) for rest. The fieldwork will be carried out in the Elmina fishing community (village) using a cross sectional study design that will employ mixed-methods to data collection and analysis. The semi-structured questionnaire will be self-administered and so participants (respondents) will be required to respond to questions from their perspective, openly and honestly. In the process, if participants wish to skip any questions they will be allowed, and if they do not wish to participate or discontinue an interview, their wish will be granted as well. A similar approach will be applied in the key informant interviews. In the case of the focus group discussions, pparticipatory learning methods will be utilized to probe for insights into settlement conditions and patterns, livelihoods, and also to understand how the fishers perceive HIV related issues, and their roles and capacities in protecting themselves from HIV. In view of this, participants shall be engaged in three exercises; a warm-up exercise, community mapping, and a role play in each FGD group. Participation in the role play will be voluntary.

Benefits/Risks of the study

Participation in the study will help in further to exploring the impacts of the approaches used in carrying out HIV related information in the study community, and find suitable ways of promoting sexual and reproductive health in fishing communities. The study findings will serve as entry point for discussion around issues related to the vulnerability, susceptibility, and the basis for formulating policies to ensure the safety and protection of fishers and fisherfolks in all fishing communities in Ghana.

There are no potential physical, social and psychological risks anticipated in participating in the study, however, owing to fact that the study bothers on sexual behaviours and relations, certain questions that may invade the privacy of participants will be asked. Participants are free to answer, skip or decline such questions if they deem them inappropriate. The issues involved in the study are varied and as a result the instruments to be used are lengthy. As indicated, the interviews for the survey will take between 45 minutes and 1 hour to complete, whilst the Key Informant Interviews and focus group discussions would take between 1 hour and 2 hours per session. To minimize stress and prevent loss of interest of participants during the interview sessions, the researcher will allow participants to have short interval breaks (15-20 minutes) for rest.

Confidentiality

The study is being carried out purely for academic reasons and so any information provided will remain strictly private, confidential and used only for academic purposes. Under no circumstance will the identity of participants be revealed to a third party.

Compensation
The study is being undertaken for academic purposes and no compensation packages have been provided for participants, however in the process of the interviews if need arises for the provision of water, the researcher will provide participants with water, to facilitate the process. Transportation cost of participants who travel purposely to meet the researcher and participate in the fieldwork shall be reimbursed.

Withdrawal from Study
Even though full participation of each participant is very important for enhancing the study outcome, participation in the study will be voluntary and participants can leave at any point, and skip any questions they do not wish to answer.

Contact for Additional Information
In case of further information, clarification on the study or in the case of research-related injury, please contact the under mentioned:

- Director of the Centre for Migration Studies University of Ghana, Dr Delali Badasu on: 0269782129 or Email: dbadasu@ug.edu.gh, or
- Prof. Mariama Awumbila of the Centre of Migration Studies, University of Ghana on: 0200994469 or Email: mawumbila@hotmail.com, and
- Prof. S. O. Kwankye of the Regional Institute for Population Studies (RIPS), University of Ghana on 0277602486 or Email: kwankyes@ug.edu.gh

If you have any questions about your rights as a research participant in this study you may contact the Administrator of the Ethics Committee for Humanities, ISSER, University of Ghana at ech@isser.edu.gh/ech@ug.edu.gh or 00233- 303-933-866.
Section C- PARTICIPANTS AGREEMENT
"I have read or have had someone read all of the above, asked questions, received answers regarding participation in this study, and am willing to give consent for me, my child/ward to participate in this study. I will not have waived any of my rights by signing this consent form. Upon signing this consent form, I will receive a copy for my personal records."

__ ____
Name of Participants

____

____
Signature or mark of participants   Date
