# Supplementary material for: Drivers and effects of fish-for-sex related single parenthood in a fishing coastal community in Ghana
Source: PLoS One. 2025 Jun 26;20(6):e0325440. doi: 10.1371/journal.pone.0325440 (PMC12200835; doi:10.1371/journal.pone.0325440)
Supplement: S5 Appendix — (DOCX) [file pone.0325440.s005.docx]

**S5 Appendix: Qualitative data**

| **Global theme** | **Organizing theme** | **Basic theme** | **Sample quote(s)** |
| --- | --- | --- | --- |
| Drivers and effects of fish-for-sex related single parenthood | Drivers of single parenthood in FFS relationships | Uncertain paternity resulting from multiple sexual partners | Some women who may have had multiple sexual partners and are unsure of the father's identity by the time they get pregnant. Consequently, they may choose to assume full responsibility for the child without considering the father's identity. (KII 1, Municipal Health Directorate)  I find it difficult to comprehend the girls' actions. How can one woman have three or more boyfriends in this small town? This is why many of them don't know the fathers of their children. All they care about is having sexual relations with fishermen after their expedition, a time when they have a lot of money to spend on women. (FGD 1, Female fisher) |
|  |  | Male partners' denial of paternity due to suspicions of promiscuity | Many women in the fishing community are unmarried and have no marital commitments, allowing them to have as many partners as they desire despite societal disapproval of such behavior. Due to this, some male fishers are aware that their partners have other partners and yet engage in sexual relations with them for sexual gratification. However, when unfortunate and unexpected pregnancies occur, some male fishers deny responsibility, accuse the women of being promiscuous, and urge them to blame their boyfriends for the pregnancy. This often leads to single female parenting as the males refuse to take responsibility for the pregnancy and the child that comes out of it. (FGD 2, Male fisher)  My boyfriend accused me of having another man several times, which led to a lack of trust in our relationship. Since then, he has not provided for me and the child. Because he has neglected his responsibilities, I am raising my child alone, even though he knows they look alike and that he is his. (FGD 3, Female fisher)  Single parenting is very common in the community. Through radio and community awareness campaigns, we provide advice on protective measures, yet only a small number of individuals heed our guidance. No matter what you tell them, many fishermen refuse to use condoms. Additionally, male fishermen often have multiple female partners, and some fish traders will engage in sexual relations with them if they have access. When these women become pregnant, the men often claim they bear no responsibility. (KII 2, Community-Based Organisation). |
|  |  | Transient fishers who become untraceable sexual partners | In this community, most fishermen are migrants from other villages. They merely trade fish and leave the community. After exchanging FFS with the women, they go before the women get pregnant. Occasionally, authorities track down some of the migrant fishers and establish settlements. However, many never return and may not even know they have children in the fishing community they had the sexual encounter. (KII 2, Department of Fisheries). |
|  |  | Neglect of children by fathers engaged in multiple partnerships | In some FFS relationships, women transition into the role of a wife without any formal marriage rites. Often, a woman is simply considered a wife by virtue of entering a sexual relationship with a man, which creates an informal partnership. This lack of formal commitment can complicate family dynamics and frequently leads to single parenting among women involved in FFS relationships. Many fishermen in these situations have multiple children across different households, making it difficult for them to provide adequate care for each one. (KII 2, Community-Based Organisation). |
|  | Effects of single-female parenting | Paternal absence | Many children in Elmina are unaware of their parents' identities. If you go to the town right now and talk to many of them, they will tell you that they don't have fathers. While this may be disheartening, it is a reality within the fishing community. (KII 2, Department of Fisheries)  Women who have sex with fishers and cannot determine the paternity of their children or identify their fathers are unable to tell their children who their fathers are, leaving these children to grow up without knowledge of who their fathers are. Many such children are found in fishing communities. (KII 5, Community Member) |
|  |  | Child developmental challenges | Most women involved in FFS relationships who become pregnant are often unable to locate their fathers or face rejection from them. As a result, they are left to manage their pregnancies and childcare alone, making it extremely challenging to provide adequate care for themselves and their children. (FGD 6, Female fisher)  Often, they (women) don't listen when you advise them, and when the men (fishers) refuse their pregnancies, then they rush to Social Welfare to complain. There are instances where we can invite some of the men to make settlements, but in many cases, we are unable to trace the men. As a result, the women struggle to support themselves, with their already impoverished family members unable to assist them. (FGD 7, Male fisher)  With fathers denying paternity and abandoning their children, these children frequently end up living with their grandparents. Mothers grappling with anger and hardship may struggle to care for them effectively. Consequently, children born into FFS relationships face significant challenges, including inadequate parental care and early sexual relationships as a means of self-support. (KII 10, KEEA official)  Regrettably, when fathers fail to identify or accept responsibility for their children's upbringing, their mothers also experience emotional disengagement from the children because of the father's actions, leading to inadequate care. Frequently, grandparents take care of the children, but due to their age and insufficient income, they often struggle to provide adequate support. (KII 9, KEEA official) |
|  |  | Maternal burden | Some girls involved in FFS relationships within the community are daughters of women who have previously engaged in FFS. Often, these girls do not know their fathers and lack parental guidance. This lack of guidance allows them to act freely, usually associating with male fishers and engaging in similar relationships. (FGD 8, Female fisher).  Honestly, parents who had their children through FFS relationships naturally put serious impediments in their lives. Since these births typically stem from a lack of love, the children often feel burdened and learn that their parents don't care about them. In extreme cases, these children may rebel by engaging in risky sexual lifestyles such as FFS and commercial sex work. (FGD 9, Male fisher). |
|  |  | Intergenerational cycle of FFS relationships | Children from FFS relationships who grow up without a paternal figure are often raised by their mothers or grandparents. The absence of a father figure can lead to emotional and social challenges as they navigate their formative years. Without the guidance and support typically provided by a father, these children may struggle to develop a sense of identity and belonging within their communities. (KII 1, Community-Based Organisation) |
